# Occurrence of Nine Grapevine Viruses in Commercial Vineyards of Mendoza, Argentina

**DOI:** 10.3390/v15010177

**Published:** 2023-01-07

**Authors:** Sebastian Gomez Talquenca, Rodrigo Alonso, Facundo Luna, Melisa Lanza Volpe, Fernando Buscema

**Affiliations:** 1 Instituto Nacional de Tecnología Agropecuaria (INTA) EEA Mendoza, Luján de Cuyo 5505, Mendoza, Argentina; 2 Instituto de Biología Agrícola de Mendoza, CONICET-Universidad Nacional de Cuyo, Chacras de Coria 5507, Mendoza, Argentina; 3 Catena Institute of Wine (CIW), Bodega Catena Zapata, Agrelo 5509, Mendoza, Argentina

**Keywords:** grapevine fanleaf virus, grapevine leafroll-associated virus, grapevine fleck virus

## Abstract

Grapevine is a widely grown fruit crop that is seriously affected by different viruses, reducing grape yield and quality, as well as threatening profitability. Vineyard disease management requires accurate identification of viral infections. This study aimed to survey the presence of ten grapevine viruses in four geographic sites in the Mendoza province of Argentina. Two hundred twenty-three composite cane samples from 1060 plants of six cultivars were collected from 26 blocks distributed across 11 vineyards. The cane samples were screened by RT-PCR for the following viruses: grapevine leafroll-associated viruses 1–4 (GLRaV 1, 2, 3, and 4), grapevine fanleaf virus (GFLV), grapevine fleck virus (GFkV), grapevine virus A (GVA) and B (GVB), grapevine rupestris stem pitting associated virus (GRSPaV), and arabis mosaic virus (ArMV). The results showed an uneven occurrence of viruses through the sampled regions, with GRSPaV being prevalent (71.1%), followed by GFLV (28.9%), GFkV (20.6%), and GLRaV-2 (14.7%). GVB was not detected. This study revealed a moderate prevalence of viruses associated with economically impactful diseases in the vineyards surveyed.

## 1. Introduction

Grapevine is the most significant fruit crop worldwide. Nearly 7.5 million hectares are cultivated in 87 countries across all five continents. Argentina stands among the top grapevine-growing countries and is fifth wine producer worldwide behind France, Spain, Italy, and the USA. Favorable local environmental and farming conditions position Argentine vineyards among the healthiest in the world [1]. However, over the last 20 years, some important pests have locally emerged or have been introduced from abroad. Viruses infecting grapevine constitute serious threats to global viticulture, causing economic losses by reducing yield, enological quality [2], and vineyard longevity [3]. 

To date, the literature describes 95 viruses that infect grapevine, many of which cause serious diseases. Strategic and effective sanitary management depends on incidence determination of the most widespread viruses. Therefore, several wine regions around the world conduct periodic and extensive surveys of viruses. In Oregon and Washington, vineyards mainly bear grapevine leafroll-associated virus 3 (GLRaV−3) and grapevine rupestris stem pitting-associated viruses (GRSPaV) [4], while in Chile, GLRaV−2 and grapevine fleck virus (GFkV) are found in greater proportion [5]. Xiao et al. [6] analyzed 17 viruses from vines sampled in Ontario (Canada) and detected high GRSPaV and GLRaV-3 predominance (87% and 48%, respectively). Even though some viral infections in Argentina were reported several decades ago [7], the economic impact was then considered almost negligible. However, during the last 15 years, increasing viral symptomatology has gained attention, and 13 new viruses were recently reported. The viruses involved in the three most deleterious grapevine diseases in the country are grapevine leafroll-associated viruses (GLRaV) 1, 2, 3 and 4 [8,9]; the rugose wood complex-associated viruses, grapevine virus A (GVA), grapevine virus E (GVE), and GRSPaV [10,11,12]; and the infectious degeneration-associated viruses, grapevine fanleaf virus (GFLV) and arabis mosaic virus (ArMV) [12]. Grapevine red blotch virus and grapevine Pinot gris virus were also recently reported [13,14]. However, at the regional level, only one study has monitored the occurrence of grapevine leafroll-associated viruses [8], while in Mendoza, no systematic survey considering major viruses has been conducted. This work aimed to determine the occurrence of ten viruses in the selected commercial vineyards of Mendoza, the most important grape-growing region in Argentina.

## 2. Materials and Methods 

**Figure 1:**
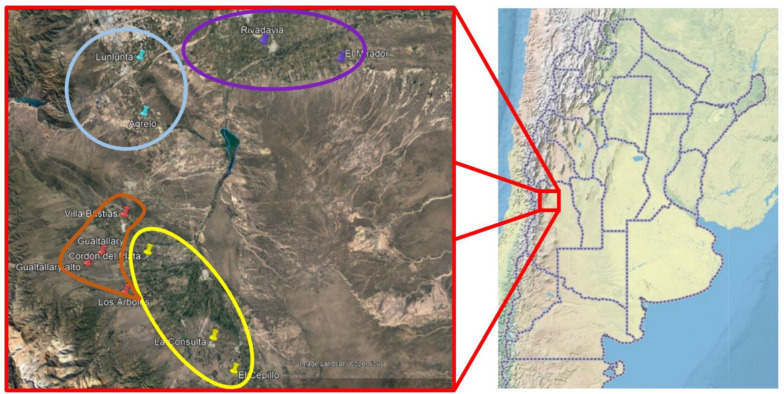
Grapevine sampling across four sites in North Mendoza. East (purple), Primera Zona (light blue), Lower Valle de Uco (yellow), and Upper Valle de Uco (orange).

Mendoza includes 70% of Argentine vineyards, distributed in four agricultural areas: North, East, Valle de Uco, and South Regions. Given the topographic and environmental features, Valle de Uco is divided into two sub-regions, the Lower and Upper Valle de Uco (Figure 1). All these regions, except the South, were assessed in this study. 

Composite sampling constitutes a suitable methodology for prevalence and incidence studies [15,16,17,18]. A total of 223 composite cane samples obtained from 1060 plants of six cultivars were collected from 26 blocks distributed among 11 vineyards (Table 1). During winter 2018, two lignified canes per vine were obtained from 4 or 5 randomly selected plants and combined into a single composite sample. According to block size, 32 to 80 plants were selected per block, resulting in a unique composite sample arrangement (Table 1). All samples were stored in plastic sealed bags at 4 °C until nucleic acid extraction and ELISA processing were carried out.

Total RNA was extracted from cambial scrapings after epidermis removal. Nearly 500 mg of cortical tissue from all canes in each composite sample were ground in liquid nitrogen in a mortar with a pestle. Then, 100 mg of the homogenized fine tissue powder was processed by the rapid CTAB method for total RNA purification, as described by Gambino et al. [19]. RNA integrity was evaluated by non-denaturing agarose gel electrophoresis and spectrophotometrical quantification. A 1 μg sample of total RNA was heat-denatured, primed with 20 pM random hexamers, and ice-chilled. First-strand cDNA was synthesized in a mix containing 20 nM dNTPs, 100 U M-MLV (RevertAid, Thermo Fisher, Waltham, MA USA), 20 U ribonuclease inhibitor (Ribolock, Thermo Fisher), and 1× RT Buffer, at 42 °C for one hour. The resulting cDNA was diluted 1:3 with nuclease-free water and immediately subjected to PCR or stored at −20 °C. The cDNA was initially amplified by PCR using 18S primers. Subsequently, samples amplifying the expected 844 bp product were subjected to ten individual PCR with specific virus primer pairs (Supplementary Table S1). In all cases, the amplification mix consisted of 2 μL of diluted cDNA, 4 pM of both forward and reverse primers, 3 nM dNTPs, 0.5 U Easy Taq DNA Polymerase (Transgen Biotech, Beijing, China), and 1X Easy Taq Buffer (Transgen Biotech), resulting in a 10 μL final volume. PCR products were resolved by electrophoresis in 1.2% agarose gels followed by ethidium bromide staining.

To confirm PCR results for GFkV, a virus not yet reported in Argentina, all positive samples of a vineyard block were analyzed by DAS-ELISA with the GFkV Reagent Set (Bioreba, Reinach, Switzerland). A total of 500 mg of cambial scrapings were grounded in 5 mL extraction buffer and processed according to the manufacturer instructions. Results were measured two hours after the addition of the substrate. A sample was considered positive if the A_405nm_ exceeded twice the average absorbance of the healthy controls.

## 3. Results

### 3.1. First Report of GFkV in Mendoza Vineyards

The expected 18S product was obtained from 218 out of 223 compositive cane samples from four regions in the Mendoza region of Argentina (Figure 1) that were tested by RT-PCR (Table 1). Forty-five samples collected from all cultivars, except for Cabernet franc, and across all regions revealed GFkV by RT-PCR. These results were confirmed by DAS-ELISA with 45 positives. Grapevine virus B (GVB) was not found in any of the 218 samples tested by RT-PCR. 

### 3.2. Nine Grapevine Viruses Are Widely Distributed among Vineyards in Mendoza

No viral amplicon was obtained in RT-PCR from 40 composite cane samples from 12 out of 26 blocks, including twenty-four Malbec composite samples (20% of the analyzed composite samples for this cv.), ten Cabernet sauvignon composite samples (29%), five Pinot noir composite samples (25%), and one Chardonnay composite sample (5%). All Cabernet franc and Aspirant Bouschet composite samples were infected with at least two viruses. GRSPaV was the most prevalent virus, detected in 71.3% of the sampled material (Table 2). After GRSPaV, GFLV showed the highest prevalence (28.9%); however, this virus was not uniformly distributed among blocks (Supplementary Table S2). Eight of the twelve GFLV-infected blocks had a 50% prevalence. A similar distribution was observed for GFkV and GRLaV-2, resulting in 6 out of 13 and 5 out of 9 blocks with more than 50% prevalence, respectively. The remaining viruses were detected in relatively few blocks, with low intra-block prevalence, except GLRaV-1 and GVA, which reached high prevalence in one and three blocks, respectively. 

**Figure 2:**
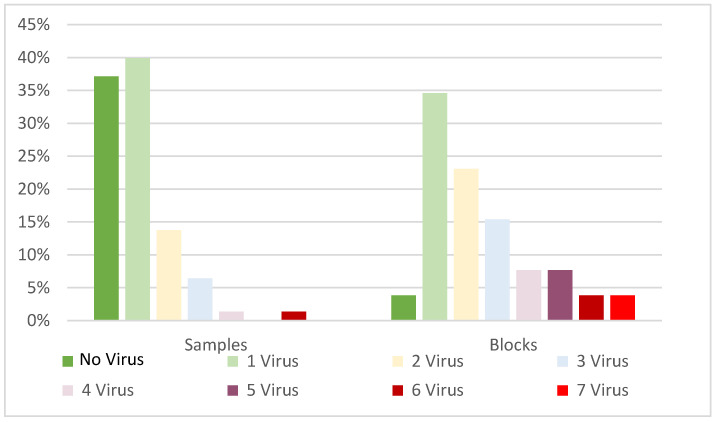
Frequency distribution of virus infections per individual sample and block (excluding GRSPaV).

Virus occurrence was uneven between the four regions. The East Region (2 to 4 °C warmer than the other regions) showed the highest prevalence of all the viruses tested, except for GFLV. On the opposite side, Upper Valle de Uco (the coolest and highest region) showed the lowest viral prevalence, particularly of GLRaV-1 and GLRaV-4, and no GLRaV-3, GVA, or ArMV (Table 3). Viral frequency distribution per sample and per block (excluding GRSPaV) showed divergent frequencies. In both cases, the prevalent modal frequency was infection with one virus. For composite samples, the second most frequent category was no virus. Finally, the second most frequent category in vineyard blocks was multiple infections (Figure 2).

## 4. Discussion

In agreement with virus surveys in most grape-growing regions of the world, the occurrence of all viruses tested was documented in this study. GRSPaV, the most frequent virus in this survey, is a ubiquitous virus in grapevines. Despite being associated with rugose wood complex and vein necrosis, its etiological role remains unclear. Considering its high prevalence and the consensual agreement on benignity [20], GRSPaV was removed from the Argentine grapevine certification scheme. Nevertheless, since most Argentine vineyards are own-rooted, local impact studies are strongly recommended. 

GVB, associated with the rugose wood complex, was not identified among the analyzed samples. Recent surveys conducted in the USA, Canada, Poland, Italy, and Pakistan reported low incidences of this virus (under 5%) in a high viral prevalence context [6,21,22,23,24,25]. A previous Chilean survey also reported very low GVB incidence (below 1%) [5]. Such findings should encourage the Argentine phytosanitary authorities to establish quarantine regulations in grapevine, preventing GVB-infected material from entering the country.

In the present study, GFLV was the second most frequent virus (28.9%). Recently, Rivadeneira et al. [26] identified GFLV in 21% of positive samples in vineyards in northwestern Argentina (the second largest wine region of the country). GFLV is transmitted by the dagger nematode *Xiphinema index* [27] but also through vegetative propagation and grafting. Our survey suggests that soil analyses should be performed for the presence of *X. index* in Argentinian grape-growing regions.

Besides GRSPaV and GFLV, the two most prevalent viruses were GFkV and GLRaV-2, in agreement with Lanza Volpe et al. [8]. Considering that both viruses lack natural vectors, no reports on natural dispersion are available; the unclear symptoms of GFkV-infected plants [28], the existence of some non-symptomatic GLRaV-2 strains [29], and the use of infected plant material likely explain the presence of these viruses in vineyards.

## 5. Conclusions

This study constitutes the first survey of grapevine viruses in selected vineyards of Mendoza, Argentina. We revealed a high prevalence of economically important virus diseases, including degeneration, leafroll, and rugose wood. 

*Planococcus ficus* is a vector of GLRaV 1, 3, and 4 [30] and is found in local vineyards. Considering that all three GLRaVs occurred in the vineyards surveyed, a combined strategy for vector control and diseased vine rouging is strongly recommended [31,32]. Otherwise, growers could face severe economic losses in terms of yield and quality, as extensively reviewed by Mannini and Digiaro [2]. A similar approach is recommended for grapevine degeneration control: diseased vine rouging and conscious *Xiphinema index* monitoring.

Finally, and considering the high viral prevalence of the surveyed blocks, the use of certified virus-tested plant material and/or laboratory testing before planting are highly encouraged sustainable viticulture practices.

## Figures and Tables

**Table 1:** Grapevine sampling in the four regions surveyed.

Region/Site	Vineyard Number	Vineyard Location	Block Number	Cultivar	Plants Sampled	Composite Samples Analyzed
Upper Valle de Uco	1	Gualtallary	1	Pinot noir	32	7
			2	Cabernet sauvignon	32	7
			3	Malbec	32	7
	2	Gualtallary Alto	4	Malbec	32	7
			5	Pinot noir	32	7
			6	Chardonnay	32	7
	3	Villa Bastias	7	Pinot noir	32	7
Lower Valle de Uco	4	Cordón del Plata	8	Chardonnay	32	7
			9	Chardonnay	32	7
	5	El Cepillo	10	Cabernet sauvignon	32	8
			11	Malbec	80	16
			12	Malbec	50	10
	6	La Consulta	13	Malbec	32	7
			14	Malbec	50	10
			15	Cabernet franc	32	7
			16	Cabernet sauvignon	50	10
			17	Malbec	50	10
			18	Cabernet sauvignon	50	10
	7	Los Arboles	19	Malbec	32	7
Primera Zona	8	Agrelo	20	Malbec	32	7
	9	Lunlunta	21	Malbec	50	10
			22	Malbec	50	10
			23	Malbec	50	10
			24	Malbec	50	10
East Region	10	Rivadavia	25	Aspirant Bouschet	32	8
	11	El Mirador	26	Aspirant Bouschet	50	10

**Table 2:** Prevalence of grapevine viruses in composite samples of different cultivars.

Cultivar	Composite Samples	GLRaV-1	GLRaV-2	GLRaV-3	GLRaV-4	GVA	GVB	GFkV	ArMV	GFLV	GRSPaV
Aspirant B.	16	38%	88%	31%	31%	19%	0%	50%	13%	31%	88%
C. Franc	7	100%	57%	0%	0%	0%	0%	0%	0%	0%	100%
C. Sauvignon	35	0%	3%	9%	11%	3%	0%	11%	0%	23%	69%
Chardonnay	20	0%	20%	0%	0%	50%	0%	35%	15%	0%	95%
Malbec	120	2%	7%	7%	0%	4%	0%	12%	12%	42%	67%
Pinot N.	20	5%	5%	0%	0%	0%	0%	60%	0%	0%	55%
Overall Prevalence	218	7.3%	14.7%	7.3%	4.1%	8.7%	0.0%	20.6%	8.7%	28.9%	71.1%

**Table 3:** Prevalence of grapevine viruses in composite samples from four regions surveyed.

Region	Composite Samples	GLRaV-1	GLRaV-2	GLRaV-3	GLRaV-4	GVA	GVB	GFkV	ArMV	GFLV	GRSPaV
Primera Zona	46	4%	2%	15%	0%	4%	0%	13%	13%	54%	87%
East Region	16	38%	88%	31%	31%	19%	0%	50%	13%	31%	88%
Upper Valle de Uco	55	2%	11%	0%	5%	0%	0%	40%	0%	16%	71%
Lower Valle de Uco	101	7%	11%	4%	1%	14%	0%	9%	11%	24%	61%

## Data Availability

Not applicable.

## Supplementary Materials

The following supporting information can be downloaded at: https://www.mdpi.com/article/10.3390/v15010177/s1: Table S1: Primers used for RT-PCR for detection of grapevine viruses. Table S2: Occurrence of grapevine viruses in the surveyed blocks.

## References

[1] Strafile D., Becerra V. (2001). Sanidad del Viñedo Argentino. IDIA XXI.

[2] Mannini F., Digiaro M. (2017). The effects of viruses and viral diseases on grapes and wine. Grapevine Viruses: Molecular Biology, Diagnostics and Management.

[3] Golino D.A., Rowhani A., Klaassen V., Sim S.T., Al Rwahnih M. Grapevine leafroll associated virus 1 effects on different grapevine rootstocks. Proceedings of the 18th International Congress on Virus and Virus-Like Diseases of Grapevine.

[4] Martin R.R., Eastwell K.C., Wagner A., Lamprecht S., Tzanetakis I.E. (2005). Survey for viruses of grapevine in Oregon and Washington. Plant Dis..

[5] Fiore N., Prodan S., Montealegre J., Aballay E., Pino A.M., Zamorano A. (2008). Survey of Grapevine viruses in Chile. J. Plant Pathol..

[6] Xiao H., Shabanian M., Moore C., Li C., Meng B. (2018). Survey for major viruses in commercial Vitis vinifera wine grapes in Ontario. Virol. J..

[7] Alcalde A.J. (1962). La dégénérescence infectieuse de la vigne dans la République Argentine.(Infectious degeneration of grapevine in Argentina). Bull. L’office Int. Vigne Vin.

[8] Lanza Volpe M., Gómez Talquenca S., Engel E., Gracia O. (2010). Incidence of Grapevine Leafroll Associated Viruses -1, -2, and -3 in Mendoza vineyards. Trop. Plant Pathol..

[9] Gómez Talquenca S., Muñoz C., Grau O., Gracia O. (2009). First description of Grapevine leafroll-associated virus 5 in Argentina and partial genome sequence. Virus Genes.

[10] Tarnowski C.G., Worlock P.A., Ulanovsky S., Gómez Talquenca S. (2002). First report of Rupestris Stem Pitting associated Virus-1 in Argentina. Plant Dis..

[11] Debat H., Zavallo D., Luna F., Moyano S., Asurmendi S., Gomez-Talquenca S. (2019). First report of grapevine virus E infecting grapevine in Argentina. J. Plant Pathol..

[12] Gracia O., Vega E., Worlock P.A. Detección de virosis de la vid en Mendoza (Argentina) con la técnica ELISA. Proceedings of the XXII Congrès de la Vigne et du vin.

[13] Debat H., Luna F., Moyano S., Zavallo D., Asurmendi S., Gomez Talquenca S. (2020). First report of Grapevine Pinot Gris Virus infecting grapevine in Argentina. J. Plant Pathol..

[14] Luna F., Debat H., Moyano S., Zavallo D., Asurmendi S., Gomez-Talquenca S. (2019). First report of grapevine red blotch virus infecting grapevine in Argentina. J. Plant Pathol..

[15] Lancaster V.A., Keller-McNulty S. (1998). A Review of Composite Sampling Methods. J. Am. Stat. Assoc..

[16] Fuchs M., Martinson T.E., Loeb G.M., Hoch H.C. (2009). Survey for the three major leafroll disease-associated viruses in Finger Lakes vineyards in New York. Plant Dis..

[17] Poojari S., Moreau D.L., Kahl D., Ritchie M., Ali S., Úrbez-Torres J.R. (2020). Disease incidence and genetic variability of economically important grapevine viruses in Nova Scotia. Can. J. Plant Pathol..

[18] Poojari S., Lowery D., Rott M., Schmidt A., Úrbez-Torres J. (2017). Incidence, distribution and genetic diversity of Grapevine red blotch virus in British Columbia. Can. J. Plant Pathol..

[19] Gambino G., Perrone I., Gribaudo I. (2008). A Rapid and effective method for RNA extraction from different tissues of grapevine and other woody plants. Phytochem. Anal..

[20] Meng B., Rowhani A. (2017). Grapevine rupestris stem pitting-associated virus. Grapevine Viruses: Molecular Biology, Diagnostics and Management.

[21] Leo G., Luison D., Grande S.B., Albanese G., Faggioli F. (2015). Grapevine Viruses’ Detection and Sanitary Selection in Grapevine Germplasm of Calabria (Southern Italy). J. Phytopathol..

[22] Jones T., Nita M. (2019). A survey of Virginia vineyards revealed high incidences of grapevine rupestris stem pitting-associated virus, grapevine red blotch virus, and two mealybug species. Plant Health Prog..

[23] Rasool S., Naz S., Rowhani A., Diaz-Lara A., Golino D.A., Farrar K.D., Al Rwahnih M. (2019). Survey of grapevine pathogens in Pakistan. J. Plant Pathol..

[24] Brannen P.M., Deom C.M., Alabi O.J., Naidu R.A. (2018). Prevalence of viruses in commercial wine grape vineyards in Georgia. Plant Health Prog..

[25] Komorowska B., Berniak H., Golis T. (2014). Detection of grapevine viruses in Poland. J. Phytopathol..

[26] Rivadeneira M., Galván M.Z., Aban M., Semke R.E., Rivadeneira J., Lanza Volpe M., Gomez Talquenca S. (2022). Survey for Major Grapevine Viruses in Commercial Vineyards of Northwestern Argentina. Plants.

[27] Andret-Link P., Laporte C., Valat L., Ritzenthaler C., Demangeat G., Vigne E., Laval V., Pfeiffer P., Stussi-Garaud C., Fuchs M. (2004). Grapevine fanleaf virus: Still a major threat to the grapevine industry. J. Plant Pathol..

[28] Sabanadzovic S., Aboughanem-Sabanadzovic N., Martelli G.P. (2017). Grapevine fleck and similar viruses. Grapevine Viruses: Molecular Biology, Diagnostics and Management.

[29] Poojari S., Alabi O.J., Naidu R.A. (2013). Molecular characterization and impacts of a strain of Grapevine leafroll-associated virus 2 causing asymptomatic infection in a wine grape cultivar. Virol. J..

[30] de Borbón C.M., Gracia O., Gómez Talquenca G.S. (2004). Mealybugs and grapevine leafroll-associated virus 3 in vineyards of Mendoza, Argentina. Am. J. Enol. Vitic..

[31] Bell V.A., Hedderley D.I., Pietersen G., Lester P.J. (2018). Vineyard-wide control of grapevine leafroll-associated virus 3 requires an integrated response. J. Plant Pathol..

[32] Pietersen G., Bell V.A., Krüger K. (2017). Management of grapevine leafroll disease and associated vectors in vineyards. Grapevine Viruses: Molecular Biology, Diagnostics and Management.

