# Supplementary material for: Occurrence of Nine Grapevine Viruses in Commercial Vineyards of Mendoza, Argentina
Source: Viruses. 2023 Jan 7;15(1):177. doi: 10.3390/v15010177 (PMC9861613; doi:10.3390/v15010177)
Supplement: Supplementary file 1 [file viruses-15-00177-s001.zip › viruses-2035415-supplementary.pdf]

Target	Primer	Primer sequence 5´-3´	Product size	Reference
18S rRNA	Forward	CGCATCATTCAAATTTCTGC	844	26
	Reverse	TTCAGCCTTGCGACCATACT		
GLRaV-2	Forward	GGTGATAACCGACGCCTCTA	543	26
	Reverse	CCTAGCTGACGCAGATTGCT		
GVB	Forward	GTGCTAAGAACGTCTTCACAGC	460	21
	Reverse	ATCAGCAAACACGCTTGAACCG		
ArMV	Forward	TGACAACATGGTATGAAGCACA	402	21
	Reverse	TATAGGGCCTTTCATCACGAAT		
GLRaV-3	Forward	TACGTTAAGGACGGGACACAGG	336	26
	Reverse	TGCGGCATTAATCTTCATTG		
GVA	Forward	GAGGTAGATATAGTAGGACCTA	272	26
	Reverse	TCGAACATAACCTGTGGCTC		
GLRaV-1	Forward	TCTTTACCAACCCCGAGATGAA	232	26
	Reverse	GTGTCTGGTGACGTGCTAAACG		
GFkV	Forward	TGACCAGCCTGCTGTCTCTA	179	26
	Reverse	TGGACAGGGAGGTGTAGGAG		
RSPaV	Forward	GGGTGGGATGTAGTAACTTTTGA	155	21
	Reverse	GCAAGTGAAATGAAAGCATCACT		
GFLV	Forward	ATGCTGGATATCGTGACCCTGT	118	26
	Reverse	GAAGGTATGCCTGCTTCAGTGG		
GLRaV-4	Forward	TGAGGTCCCATGTCATGAC	457	26
	Reverse	CCTCAATCTRTTSACCAAYTCAC		

Suplementary Table S1. Primers used for RT-PCR for detection of grapevine viruses.

Region	Vineyard Location	Cultivar	GLRaV-1	GLRaV-2	GLRaV-3	GLRaV-4	GVA	GVB	GFkV	ArMV	GFLV	RSPaV
Primera Zona	Agrelo	Malbec	0%	0%	0%	0%	0%	0%	0%	0%	100%	100%
Primera Zona	Lunlunta	Malbec	20%	0%	50%	0%	20%	0%	60%	60%	0%	100%
Primera Zona	Lunlunta	Malbec	0%	0%	10%	0%	0%	0%	0%	0%	40%	100%
Primera Zona	Lunlunta	Malbec	0%	0%	10%	0%	0%	0%	0%	0%	80%	60%
Primera Zona	Lunlunta	Malbec	0%	11%	0%	0%	0%	0%	0%	0%	67%	78%
Upper Valle de Uco	Gualtallary	C.sauvignon	0%	0%	0%	43%	0%	0%	29%	0%	14%	57%
Upper Valle de Uco	Gualtallary	Malbec	0%	0%	0%	0%	0%	0%	0%	0%	29%	57%
Upper Valle de Uco	Gualtallary	Pinot noir	0%	0%	0%	0%	0%	0%	100%	0%	0%	100%
Upper Valle de Uco	Gualtallary Alto	Chardonnay	0%	0%	0%	0%	0%	0%	14%	0%	0%	86%
Upper Valle de Uco	Gualtallary Alto	Malbec	0%	0%	0%	0%	0%	0%	0%	0%	86%	100%
Upper Valle de Uco	Gualtallary Alto	Pinot noir	0%	0%	0%	0%	0%	0%	29%	0%	0%	71%
Upper Valle de Uco	Villa Bastias	Pinot noir	14%	14%	0%	0%	0%	0%	57%	0%	0%	0%
Lower Valle de Uco	Cordon del Plata	Chardonnay	0%	67%	0%	0%	67%	0%	33%	33%	0%	100%
Lower Valle de Uco	Cordon del Plata	Chardonnay	0%	0%	0%	0%	86%	0%	57%	14%	0%	100%
Lower Valle de Uco	El Cepillo	C.sauvignon	0%	13%	13%	0%	0%	0%	13%	0%	0%	75%
Lower Valle de Uco	El Cepillo	Malbec	0%	0%	0%	0%	0%	0%	0%	0%	56%	19%
Lower Valle de Uco	El Cepillo	Malbec	0%	0%	0%	0%	0%	0%	0%	0%	0%	0%
Lower Valle de Uco	La Consulta	C.franc	100%	57%	0%	0%	0%	0%	0%	0%	0%	100%
Lower Valle de Uco	La Consulta	C.sauvignon	0%	0%	10%	0%	0%	0%	0%	0%	0%	40%
Lower Valle de Uco	La Consulta	C.sauvignon	0%	0%	10%	10%	10%	0%	10%	0%	70%	100%
Lower Valle de Uco	La Consulta	Malbec	0%	0%	0%	0%	14%	0%	0%	29%	0%	71%
Lower Valle de Uco	La Consulta	Malbec	0%	20%	10%	0%	20%	0%	10%	60%	30%	40%
Lower Valle de Uco	La Consulta	Malbec	0%	0%	0%	0%	0%	0%	0%	0%	50%	100%
Lower Valle de Uco	Los Arboles	Malbec	0%	71%	0%	0%	0%	0%	100%	0%	0%	100%
East Region	El Mirador	Aspirant B.	0%	78%	22%	11%	0%	0%	89%	0%	0%	78%
East Region	Rivadavia	AspirantB.	86%	100%	43%	57%	43%	0%	0%	29%	71%	100%

Suplementary Table S2. Occurrence of grapevine viruses in the surveyed blocks.
